# Supplementary material for: Glucocorticoid receptor wields chromatin interactions to tune transcription for cytoskeleton stabilization in podocytes
Source: Commun Biol. 2021 Jun 3;4:675. doi: 10.1038/s42003-021-02209-8 (PMC8175753; doi:10.1038/s42003-021-02209-8)
Supplement: Supplementary file 2 — Supplementary Information [file 42003_2021_2209_MOESM2_ESM.pdf]

Supplementary Fig. 1: Morphology and podocyte marker expression of hPC during cell culture.

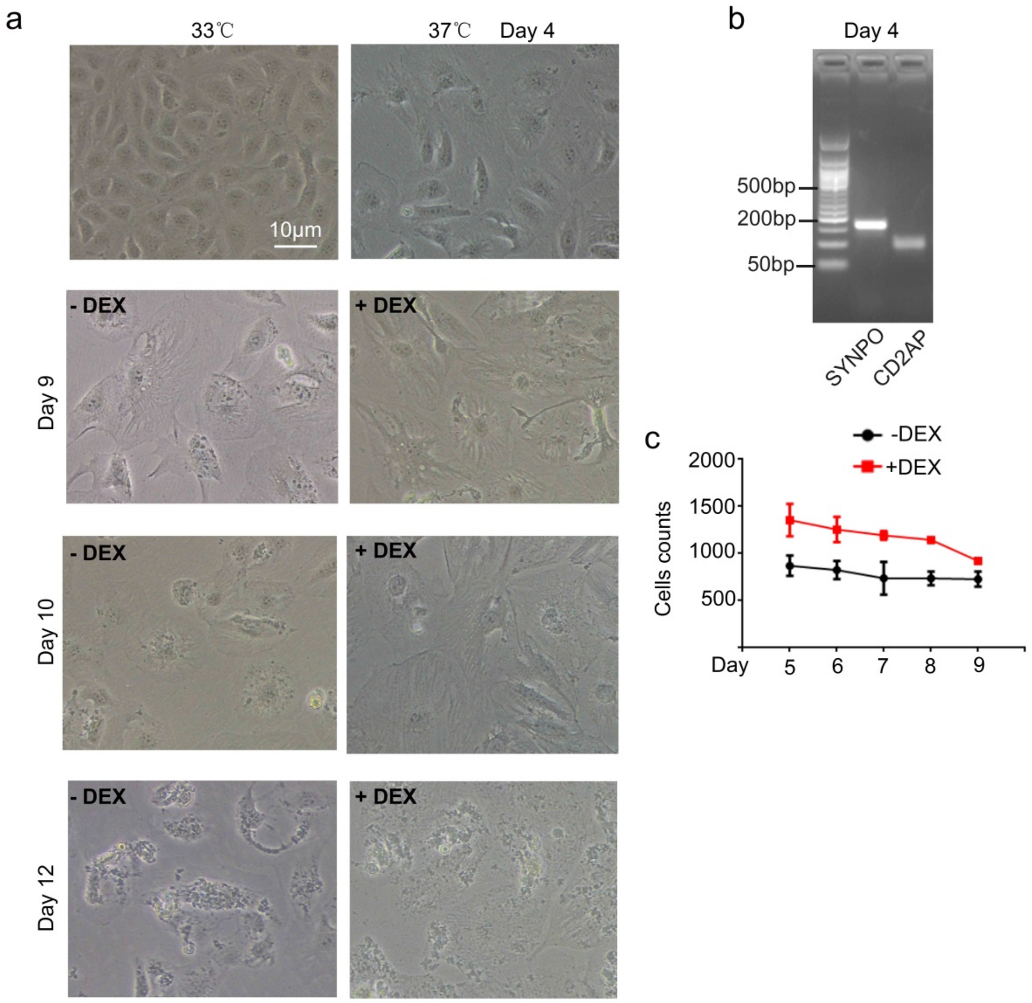

a Representative pictures of hPC morphology at different days during differentiation. **b** Marker expression in hPC differentiated at day4. **c** Cell numbers counted during the hPC differentiation. Results from 3 replications. Error bars are standard deviation (SD).

Supplementary Fig. 2: Comparison of GR profiles in hPC treated with DEX (this study) or prednisolone (McCaffrey et al.).

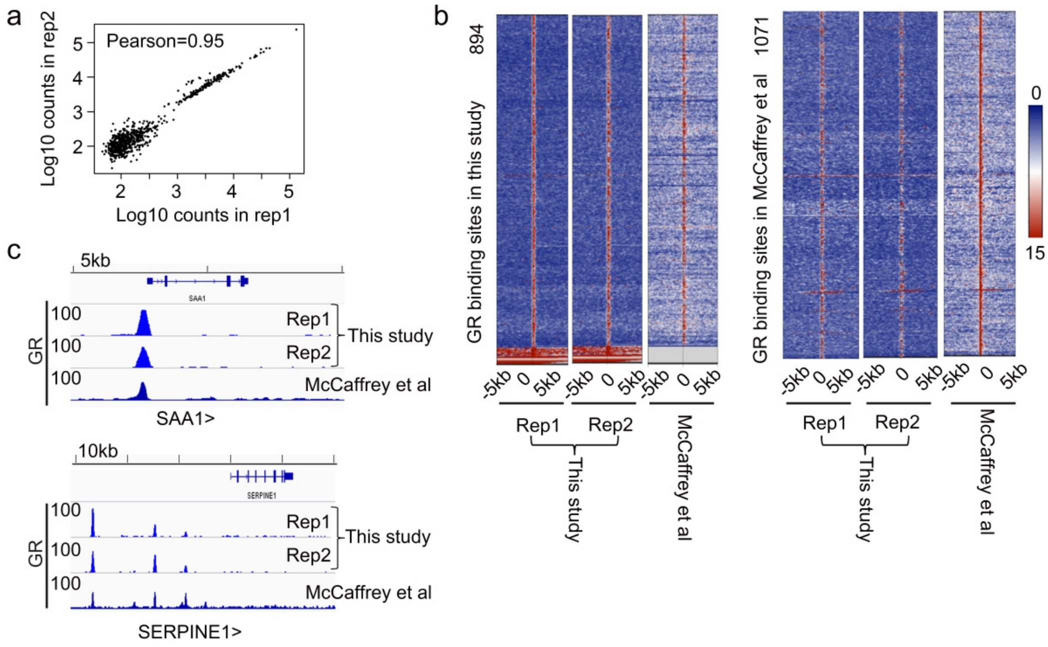

a The correlation between two replicates of GR ChIP-seq in this study. **b** Heatmaps of ChIP-seq signal from the three profiles at GR peaks called from this study (left) or from McCaffrey et al. study (right). **c** Genome browser view of GR ChIP-seq signals at SAA1 and SERPINE1 loci.

Supplementary Fig. 3: Comparison of GR binding between hPC and K562, A549, Hela or BEAS-2B.

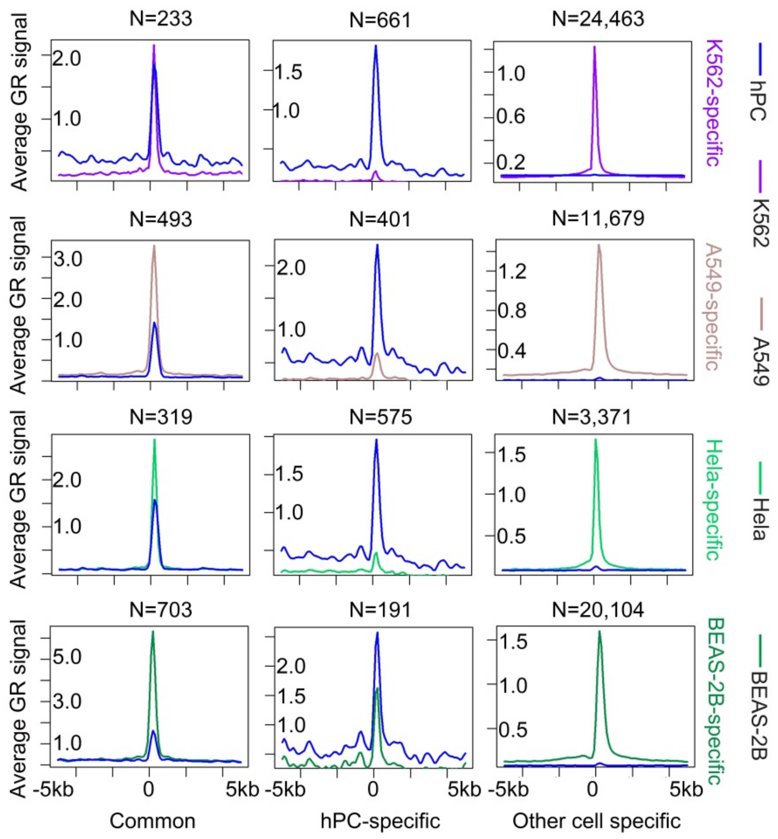

Average plot of GR ChIP signals in hPC and other cells in three groups: shared sites, hPC specific sites and other cell specific sites. N represents the number of sites in each group.

Supplementary Fig. 4: Replications of H3K27ac ChIP-seq without or with DEX treatment.

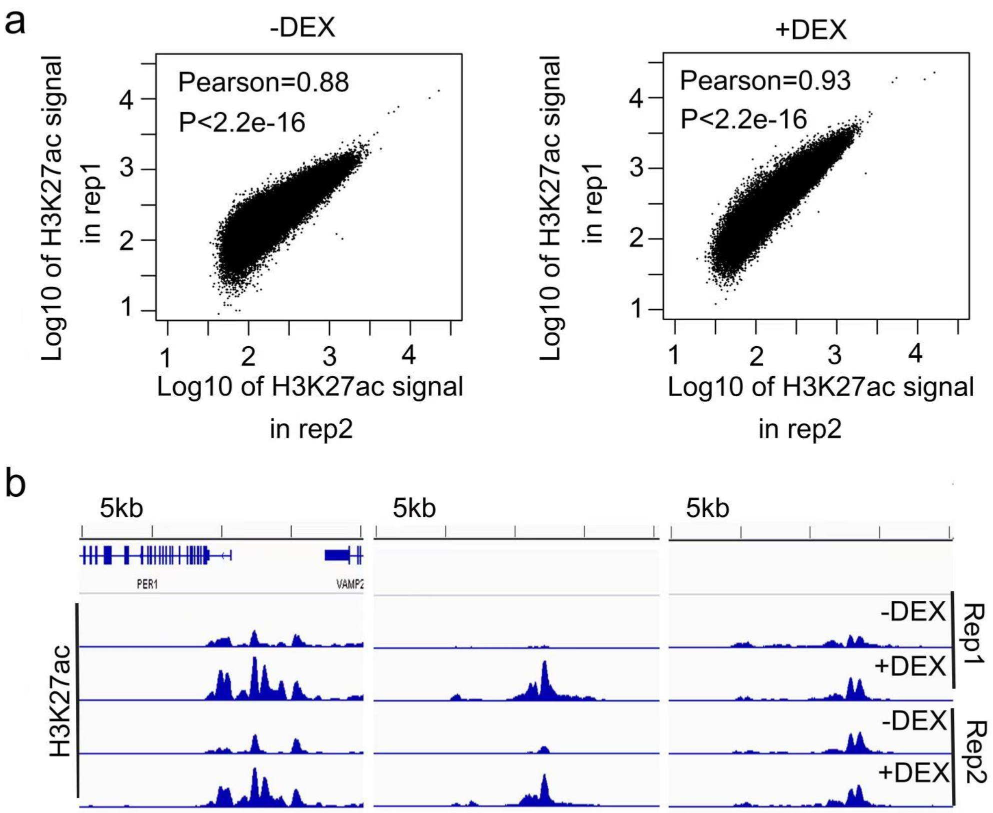

a Point plot shows log10 of H3K27ac signal in two replicates without or with DEX treatment. **b** Examples shows the H3K27ac binding profiles without or with DEX treatment in two replicates. The regions are the same as Fig. 2c.

Supplementary Fig. 5: H3K27ac binding profiles at GR sites.

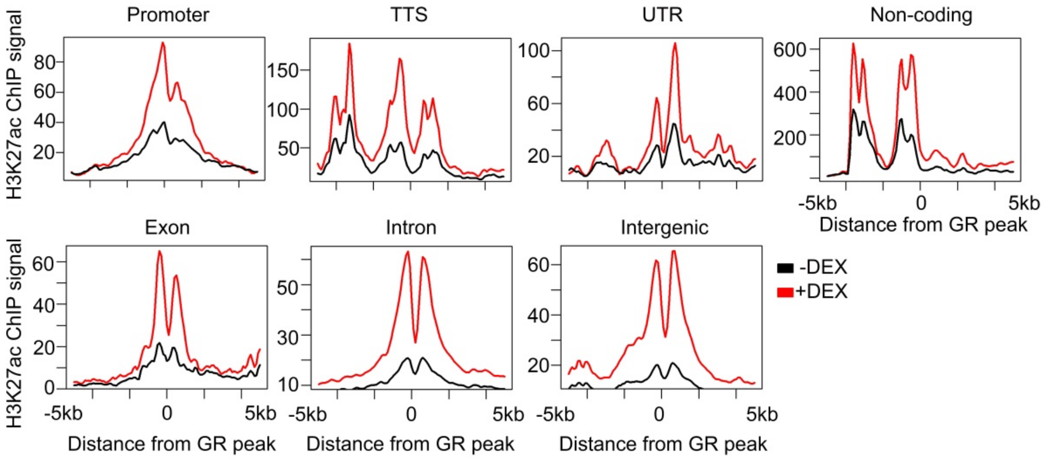

Average plots show H3K27ac signal at GR sites located in various genomic regions.

Supplementary Fig. 6: Transcription dynamics after DEX treatment.

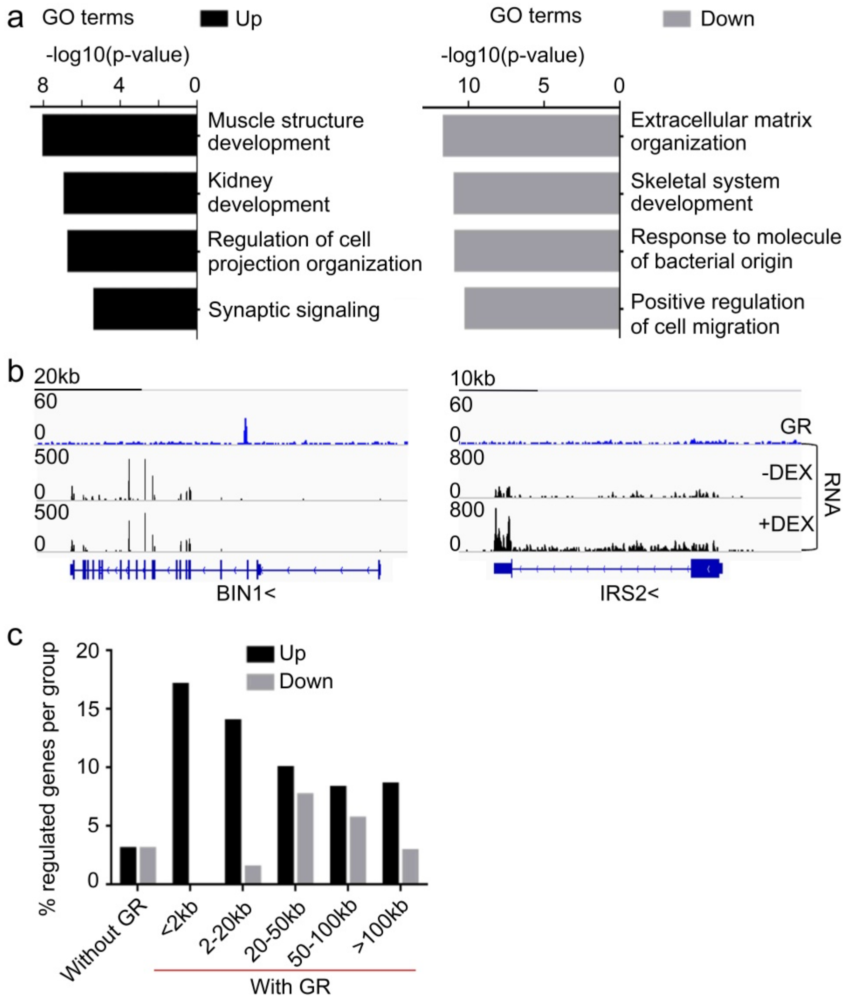

a Gene ontology analysis of DEX-responsive genes (P<0.05). **b** Genome browser view of GR binding and RNA-seq signals at loci of BIN1and IRS2. BIN1 did not change transcription though harboring a GR binding, while IRS2 increased transcription without any GR binding. **c** Percentage of differential regulated genes (p<0.05) without or with GR binding at different distance.

Supplementary Fig. 7: 3D map construction and dynamic loops analysis.

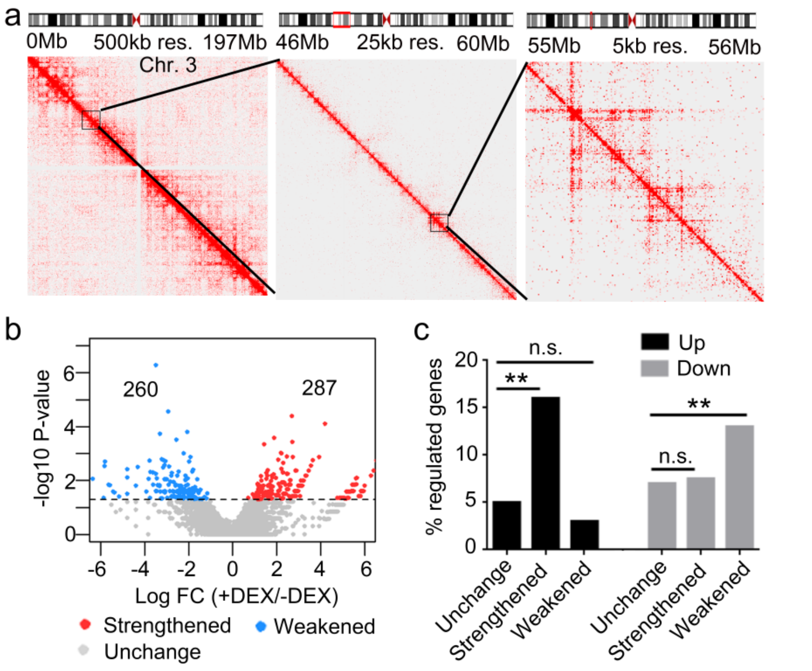

a Example of chromatin interaction maps at 500kb, 25kb and 5kb resolution for Chr3. **b** LogFC and –log10 (P-value) of loops in hPC with or without DEX treatment. Red points represent the strengthened interaction frequency ($p<0.05$, log(foldchange)>0), blue points represents the weakened interaction frequency ($p<0.05$, log(foldchange)<0) and grey points represents unchanged interaction frequency ($p>0.05$). **c** The proportion of differential expressed genes in unchanged, strengthened and weakened loops. For up-regulated group, 'Unchange' vs 'Strengthened' p=0.00005, 'Unchange' vs 'Weakended' p=11. For down-regulated group, 'Unchange' vs 'Strengthened' p=0.72, 'Unchange' vs 'Weakended' p=0.009. Chi-square test, *P<0.05, **P<0.01, n.s. no significance. Black bar represents up-regulated genes ($p<0.05$, log(foldchange)>0) and grey bar represents down-regulated genes ($p<0.05$, log(foldchange)<0).

Supplementary Fig. 8: Features of GR or non-GR loops in hPC and A549.

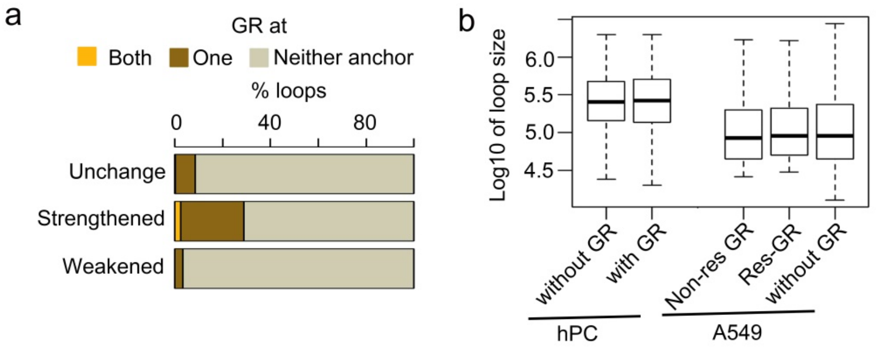

a The proportion of various loops overlapped with GR at both, one, or neither anchors in hPC. **b** Size of loops in various groups in hPC and A549. Center line, median; box limits, upper and lower quantiles.

Supplementary Fig. 9: H3K27ac signal at SEs before and after DEX treatment.

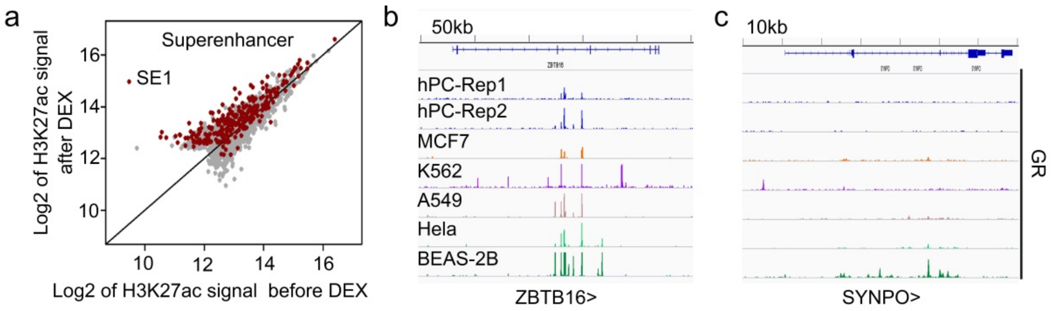

a Dot plot of H3K27ac signal in hPC before or after DEX treatment for all SEs (grey points), or for GR associated SEs (dark red points) in hPC. **b,c** GR binding profile at ZBTB16 (b) and SYNPO loci (c) in different cell types.
