# Supplementary material for: Glucocorticoid receptor wields chromatin interactions to tune transcription for cytoskeleton stabilization in podocytes
Source: Commun Biol. 2021 Jun 3;4:675. doi: 10.1038/s42003-021-02209-8 (PMC8175753; doi:10.1038/s42003-021-02209-8)
Supplement: Supplementary file 3 — Description of Additional Supplementary Files [file 42003_2021_2209_MOESM3_ESM.pdf]

Description of Additional Supplementary Files

File name: Supplementary Data 1

Description: Pairwaise comparison of GR ChIP-seq.

File name: Supplementary Data 2

Description: 6-cell comparison.

File name: Supplementary Data 3

Description: super-enhancer list.

File name: Supplementary Data 4

Description: Chromatin interaction coordinations.

File name: Supplementary Data 5

Description: Go analysis of GR-SE.

File name: Supplementary Data 6

Description: ZBTB16 expression in patients.

File name: Supplementary Data 7

Description: Density of F-actin.

File name: Supplementary Data 8

Description: qPCR results.
