# Supplementary material for: Glucocorticoid receptor wields chromatin interactions to tune transcription for cytoskeleton stabilization in podocytes
Source: Commun Biol. 2021 Jun 3;4:675. doi: 10.1038/s42003-021-02209-8 (PMC8175753; doi:10.1038/s42003-021-02209-8)
Supplement: Supplementary file 12 — Reporting Summary [file 42003_2021_2209_MOESM12_ESM.pdf]

nature research

Corresponding author(s): Zhihong Liu, Jingping Yang

Last updated by author(s): Apr 30, 2021

Reporting Summary

Nature Research wishes to improve the reproducibility of the work that we publish. This form provides structure for consistency and transparency in reporting. For further information on Nature Research policies, see our Editorial Policies and the Editorial Policy Checklist.

Statistics

For all statistical analyses, confirm that the following items are present in the figure legend, table legend, main text, or Methods section.

n/a	Confirmed	
☐	☒	The exact sample size (*n*) for each experimental group/condition, given as a discrete number and unit of measurement
☐	☒	A statement on whether measurements were taken from distinct samples or whether the same sample was measured repeatedly
☐	☒	The statistical test(s) used AND whether they are one- or two-sided<br>*Only common tests should be described solely by name; describe more complex techniques in the Methods section.*
☒	☐	A description of all covariates tested
☒	☐	A description of any assumptions or corrections, such as tests of normality and adjustment for multiple comparisons
☒	☐	A full description of the statistical parameters including central tendency (e.g. means) or other basic estimates (e.g. regression coefficient) AND variation (e.g. standard deviation) or associated estimates of uncertainty (e.g. confidence intervals)
☐	☒	For null hypothesis testing, the test statistic (e.g. *F*, *t*, *r*) with confidence intervals, effect sizes, degrees of freedom and *P* value noted<br>*Give P values as exact values whenever suitable.*
☒	☐	For Bayesian analysis, information on the choice of priors and Markov chain Monte Carlo settings
☒	☐	For hierarchical and complex designs, identification of the appropriate level for tests and full reporting of outcomes
☒	☐	Estimates of effect sizes (e.g. Cohen's *d*, Pearson's *r*), indicating how they were calculated

Our web collection on statistics for biologists contains articles on many of the points above.

Software and code

Policy information about availability of computer code

Data collection	N/A
Data analysis	HISAT2 (2.0.5); Stringtie(1.3.3); Bowtie2 (2.3.0); MACS2; HiC-Pro (2.8.0); Hichipper; Juicebox; Diffloop (1.10.0); ROSE; ImageJ; DESeq2 (1.22.2); edge R (3.24.3); R (3.5.1)

For manuscripts utilizing custom algorithms or software that are central to the research but not yet described in published literature, software must be made available to editors and reviewers. We strongly encourage code deposition in a community repository (e.g. GitHub). See the Nature Research guidelines for submitting code & software for further information.

Data

Policy information about availability of data

All manuscripts must include a data availability statement. This statement should provide the following information, where applicable:

- Accession codes, unique identifiers, or web links for publicly available datasets
- A list of figures that have associated raw data
- A description of any restrictions on data availability

RNA-seq, ChIP-seq and HiChIP-seq data used in this study have been submitted to NCBI Gene Expression Omnibus (GEO; http://www.ncbi.nlm.nih.gov/geo/) under accession number GSE117888. Secure token for GSE117888 is 'ijirooowdpcrdc'.

Field-specific reporting

Please select the one below that is the best fit for your research. If you are not sure, read the appropriate sections before making your selection.

- [x] Life sciences
- [ ] Behavioural & social sciences
- [ ] Ecological, evolutionary & environmental sciences

For a reference copy of the document with all sections, see nature.com/documents/nr-reporting-summary-flat.pdf

Life sciences study design

All studies must disclose on these points even when the disclosure is negative.

Sample size	No sample-size calculation was performed.
Data exclusions	No data were excluded from the analysis.
Replication	The sequencing data were performed with two replications. All attempts at replication were successful.
Randomization	Cells from the same aliquot were used for each condition.
Blinding	Investigators analyzed the data with knowledge of treatment of cells.

Reporting for specific materials, systems and methods

We require information from authors about some types of materials, experimental systems and methods used in many studies. Here, indicate whether each material, system or method listed is relevant to your study. If you are not sure if a list item applies to your research, read the appropriate section before selecting a response.

Materials & experimental systems

n/a	Involved in the study	
	x	Antibodies
	x	Eukaryotic cell lines
x		Palaeontology and archaeology
x		Animals and other organisms
x		Human research participants
x		Clinical data
x		Dual use research of concern

Methods

n/a	Involved in the study	
	x	ChIP-seq
x		Flow cytometry
x		MRI-based neuroimaging

Antibodies

Antibodies used	GR, Santa Cruz Biotechnology, sc1003X; H3K27ac, Abcam, ab4729; ZBTB16, R&D Systems, MAB2944.
Validation	All the used antibodies are commercial antibodies are validated by the antibody manufacturer and have been used in published references.

Eukaryotic cell lines

Policy information about cell lines

Cell line source(s)	The immortalized human podocytes were provided by Dr. Saleem M (University of Bristol, United Kingdom).
Authentication	The cell line were authenticated by checking the podocyte marker after differentiation.
Mycoplasma contamination	The cell lines were not tested for mycoplasma contamination.
Commonly misidentified lines (See ICLAC register)	N/A

ChIP-seq

Data deposition

☒ Confirm that both raw and final processed data have been deposited in a public database such as GEO.

☒ Confirm that you have deposited or provided access to graph files (e.g. BED files) for the called peaks.

Data access links<br>*May remain private before publication.*	GSE117888 with security token 'ijirooowdpcrdc'.
Files in database submission	GSM3314496_GR-ChIP-hg38_peaks.narrowPeak.gz<br>GSM3314496_GR-ChIP-hg38_treat_pileup.bedgraph.gz<br>GSM3314496_GR.bedgraph.gz<br>GSM3314496_GR.narrowPeak.gz<br>GSM3314497_H3K27ac-CTL-ChIP-hg38_peaks.narrowPeak.gz<br>GSM3314497_H3K27ac-CTL-ChIP-hg38_treat_pileup.bedgraph.gz<br>GSM3314497_H3K27ac-CTL.bedgraph.gz<br>GSM3314497_H3K27ac-CTL.broadPeak.gz<br>GSM3314498_H3K27ac-DEX-ChIP-hg38_peaks.narrowPeak.gz<br>GSM3314498_H3K27ac-DEX-ChIP-hg38_treat_pileup.bedgraph.gz<br>GSM3314498_H3K27ac-DEX.bedgraph.gz<br>GSM3314498_H3K27ac-DEX.broadPeak.gz<br>GSM3314500_RNAseq-CTL1.gtf.gz<br>GSM3314501_RNAseq-CTL2.gtf.gz<br>GSM3314502_RNAseq-DEX1.gtf.gz<br>GSM3314503_RNAseq-DEX2.gtf.gz<br>GSM3444788_DEX_allValidPairs.hic<br>GSM3444788_H3K27ac-DEX1-HiChIP-hg38_allValidPairs.hic<br>GSM3444789_H3K27ac-DEX2-HiChIP-hg38_allValidPairs.hic<br>GSM3444790_CTL_allValidPairs.hic<br>GSM3444790_H3K27ac-CTL1-HiChIP-hg38_allValidPairs.hic<br>GSM3444791_H3K27ac-CTL2-HiChIP-hg38_allValidPairs.hic<br>GSM5008647_GR2-ChIP-hg38_peaks.narrowPeak.gz<br>GSM5008647_GR2-ChIP-hg38_treat_pileup.bedgraph.gz<br>GSM5008647_GR2-ChIP-seq_peaks.narrowPeak.gz<br>GSM5008647_GR2-ChIP-seq_treat_pileup.bedgraph.gz<br>GSM5008648_GR-1h-ChIP-hg38_peaks.narrowPeak.gz<br>GSM5008648_GR-1h-ChIP-hg38_treat_pileup.bedgraph.gz<br>GSM5008648_GR-1h-ChIP-seq_peaks.narrowPeak.gz<br>GSM5008648_GR-1h-ChIP-seq_treat_pileup.bedgraph.gz<br>GSM5008649_H3K27ac-CTL2--ChIP-hg38_peaks.narrowPeak.gz<br>GSM5008649_H3K27ac-CTL2-ChIP-hg38_treat_pileup.bedgraph.gz<br>GSM5008649_H3K27ac-CTL2-ChIP-seq_peaks.narrowPeak.gz<br>GSM5008649_H3K27ac-CTL2-ChIP-seq_treat_pileup.bedgraph.gz<br>GSM5008650_H3K27ac-DEX2--ChIP-hg38_treat_pileup.bedgraph.gz<br>GSM5008650_H3K27ac-DEX2-ChIP-hg38_peaks.narrowPeak.gz<br>GSM5008650_H3K27ac-DEX2-ChIP-seq_peaks.narrowPeak.gz<br>GSM5008650_H3K27ac-DEX2-ChIP-seq_treat_pileup.bedgraph.gz
Genome browser session<br>(e.g. UCSC)	N/A

Methodology

Replicates	Two biological replications for each antibody or condition. The correlations between replications are larger than 0.9.
Sequencing depth	All the ChIP-seq libraries were sequenced by pair end 150bp mode. The total number of reads and uniquely mapped reads were 62M and 48M for input, 28M and 21 M for GR ChIP-seq replication 1, 16M and 14M for GR ChIP-seq replication 2, 34M and 32M for H3K27ac (-Dex) ChIP-seq replication 1, 26M and 24M for H3K27ac (-Dex) ChIP-seq replication 2, 34M and 31M for H3K27ac (+Dex) ChIP-seq replication 1, 15M and 14M for H3K27ac (+Dex) ChIP-seq replication 2.
Antibodies	GR, Santa Cruz Biotechnology, sc1003X; H3K27ac, Abcam, ab4729.
Peak calling parameters	Bowtie2 was used for mapping and MACS2 was used for peak calling. The index file we used is H. sapiens, hg19 provided by Bowtie2. The q value in MACS2 is 0.01 for GR ChIP-seq and 0.05 for H3K27ac ChIP-seq.
Data quality	Two replications were performed for each antibody and for each condition. The replications correlate well. The number of peaks at

	FDR 5% and 5-fold enrichment are 772 for GR ChIP-seq replication 1, 670 for GR ChIP-seq replication 2, 63562 for H3K27ac (-Dex) ChIP-seq replication 1, 55698 for H3K27ac (-Dex) ChIP-seq replication 2, 60840 for H3K27ac (+Dex) ChIP-seq replication 1, 52043 for H3K27ac (+Dex) ChIP-seq replication 2.
Software	Bowtie2 (2.3.0); MACS2; ROSE; R (3.5.1)
